# NEDD4L inhibits epithelial‐mesenchymal transition in gastric cancer by mediating BICC1 ubiquitination

**DOI:** 10.1002/kjm2.12924

**Published:** 2024-12-24

**Authors:** Shaoyi Duan, Zhiliang Tian, Rong Hu, Heng Long

**Affiliations:** ^1^ Hunan University of Medicine Huaihua Hunan Province People's Republic of China

**Keywords:** BICC1, epithelial‐mesenchymal transition, gastric cancer, NEDD4L, PI3K/AKT

## Abstract

Epithelial‐mesenchymal transition (EMT) is a critical stage in the metastasis of gastric cancer (GC). Further clarification of the EMT process in GC is still needed. This study examined the effects of the NEDD4L/BICC1 axis on GC proliferation and the EMT process. Thirty GC patients were enrolled in this study to assess the expression of BICC1 and NEDD4L in tumor samples. A xenograft tumor model in mice was created to investigate BICC1's function in vivo. The proliferation, migration, and invasion of GC cells were evaluated using colony formation, transwell, and wound healing assays. Western blot determined the expression levels of EMT‐associated proteins. Co‐immunoprecipitation (Co‐IP) elucidated the mechanism by which NEDD4L regulates BICC1. BICC1 was found to be overexpressed in tumors. Additionally, BICC1 knockdown inhibited the growth of GC cells in vivo and prevented their migration, invasion, proliferation, and EMT. Furthermore, BICC1 activated the PI3K/AKT pathway, which facilitated cancer progression. Tumor tissues and GC cells exhibited low expression levels of NEDD4L. Conversely, NEDD4L overexpression promoted the ubiquitination and degradation of BICC1 protein, thereby inhibiting GC cell proliferation, migration, invasion, and EMT processes. Our study demonstrated that NEDD4L acts as a tumor suppressor in GC, while BICC1 functions as a pro‐tumorigenic factor. The NEDD4L/BICC1 axis plays a significant role in the metastasis and progression of GC.

**Abbreviations**
AKT: protein kinase B
BICC1: BicC family RNA binding protein 1
CHX: cycloheximide
Co‐IP: co‐immunoprecipitation
DAB: 3, 3′‐diaminobenzidine
EMT: epithelial‐mesenchymal transition
GC: gastric cancer
IHC: immunohistochemistry
NEDD4L: NEDD4 like E3 ubiquitin protein ligase
PI3K: phosphatidylinositol 3‐kinase
RT‐qPCR: quantitative reverse transcription PCR
WB: western blot

## 1 INTRODUCTION

Gastric cancer (GC) is one of the most prevalent cancers worldwide. Owing to difficulties in detecting GC at early stages, most patients face a poor prognosis upon diagnosis.^1^ Among all cancer types, GC has the third‐highest incidence and mortality rates in China, accounting for approximately about 44.0% and 48.6% of GC‐related deaths globally.^2^ Metastasis is a significant factor contributing to the difficulty in treating GC and the high rates of recurrence among patients.^3^, ^4^ The most common pathway for GC metastasis is lymph node involvement. Given the high heterogeneity of GC, the mechanisms underlying its metastasis are complex and poorly understood.

The term “epithelial‐mesenchymal transition” (EMT) refers to the gradual transformation of epithelial cells into cells that exhibit characteristics of mesenchymal tissue. EMT is associated with cancer development, as it enhances the invasiveness and motility of cancer cells, thereby facilitating their spread.^5^ The occurrence of EMT is closely linked to the metastatic process of GC, and inhibiting EMT can suppress the metastasis of GC cells. For example, the knockdown of Flotillin‐1 has been shown to prevent the metastasis of GC cells by reducing the production of Snail, a protein involved in EMT.^6^ Additionally, PRSS2 has been found to enhance MMP‐9‐mediated EMT, which further promotes the motility and invasion of GC cells.^7^ Therefore, EMT may play a critical role in initiating the malignant manifestations of metastasis in cancer.

Bicaudal C homolog 1 (BICC1) is an RNA‐binding protein that post‐transcriptionally regulates a significant amount of mRNA in the cytoplasm.^8^ Notably, BICC1 plays a crucial role in tumors biology. Researchers have discovered that BICC1 is highly expressed in oral cancer, where it promotes tumor growth by enhancing cell survival and preventing apoptosis. In pancreatic cancer,^9^ BICC1 facilitates metastasis by binding to the mRNA of LCN2, thereby increasing LCN2 expression.^10^ Furthermore, BICC1 is significantly expressed in GC and shows a strong correlation with the invasion depth and tumor grading in GC patients,^11^ suggesting its involvement in GC progression. BICC1 is also associated with lymph node metastasis in pancreatic cancer patients, and is enriched in the major EMT pathway, promoting tumor progression.^12^ Additionally, PI3K/AKT signaling pathway, which is related to EMT, mediates the downregulation of mesenchymal markers and the loss of the epithelial marker E‐cadherin.^5^, ^13^, ^14^ BICC1 is significantly enriched in the PI3K/AKT signaling pathway in colon adenocarcinoma.^15^ In conclusion, we hypothesize that BICC1 may promote PI3K/AKT‐induced EMT in GC.

NEDD4L is an E3 ubiquitin ligase that has been reported in several studies to inhibit cancer progression by promoting the ubiquitin‐mediated degradation of downstream proteins. For example, NEDD4L prevents the proliferation of prostate cancer cells by regulating PHF8 through the ubiquitin‐proteasome system.^16^ Additionally, breast cancer cells undergo autophagic cell death due to the ubiquitination and degradation of YAP1, a process facilitated by NEDD4L.^17^ Furthermore, Gao et al. found a negative correlation between poor patient prognosis and decreased NEDD4L expression in GC tissues.^18^ Moreover, NEDD4L negatively regulates the EMT process in tumors. For example, the downregulation of NEDD4L expression in lung cancer cells enhances TGF‐*β*‐induced EMT to increase metastatic progression.^19^ NEDD4L may also possess anticancer properties in renal carcinoma and has the potential to reverse the EMT process both in vitro and in vivo.^20^ However, reports on NEDD4L's role in the progression of GC are limited, and the precise mechanism remains unknown. Based on predictions from the UbiBrowser database, we hypothesize that BICC1 may serve as a ubiquitination substrate for NEDD4L, suggesting that NEDD4L could inhibit the EMT process in GC cells through the ubiquitin‐mediated degradation of BICC1.

We hypothesized that NEDD4L decreased tumor growth by ubiquitinating and degrading BICC1 in GC cells, thereby inhibiting the PI3K/AKT‐mediated EMT process based on the above findings.

## 2 MATERIALS AND METHODS

### 2.1 Patients

We recruited 30 patients with GC at Hunan University of Medicine and excluded those who had received adjuvant chemotherapy, radiotherapy, or immunotherapy prior to the surgical removal of the tumor. Samples of GC and matched para‐cancerous tissues were collected immediately following surgical excision. The samples were stored in a refrigerator at −80°C. Written informed consent was obtained from each patient, and the study was approved by the Ethics Committee of Hunan University of Medicine.

### 2.2 Cell culture

In this study, human gastric mucosa cell line GES‐1, and GC cell lines AGS, MKN45, HGC‐27, and NCI‐N87 were employed. The ATCC (Manassas, VA, USA) provided the GES‐1 and AGS cells; Shanghai Chinese Academy of Sciences Cell Bank (Shanghai, China) provided MKN45, HGC‐27, and NCI‐N87. GES‐1 cells were cultured in DMEM/F‐12 1:1 medium, while the cells (AGS, MKN45, HGC‐27, and NCI‐N87) were grown in RPMI‐1640 medium. 1% penicillin/streptomycin and 10% fetal bovine serum (FBS, Gibco, Gaithersburg, MD, USA) were added to the cell culture mix as supplements, and cells were cultured at room temperature and 5% CO_2_.

### 2.3 Cell transfection

For transfection, we utilized shRNA (sh‐BICC1) and a plasmid vector (OE‐BICC1) to knock down or overexpress BICC1, while the overexpression plasmid vector OE‐NEDD4L was employed to overexpress NEDD4L. These shRNAs and plasmid vectors were designed by GenePharma (Shanghai, China). Subsequently, Lipofectamine™ 3000 (ThermoFisher, Carlsbad, CA, USA) was used to transfect AGS and MKN45 cells for 48 h with the above plasmids or shRNAs. After transfection, the cells were washed with PBS and cultured in fresh complete medium for further analysis. For the agonist‐treated group, AGS and MKN45 cells were pretreated with 20 μM of the PI3K/Akt agonist 740Y‐P (Sigma‐Aldrich, St. Louis, MO, USA) for 24 h prior to shRNA transfection.

### 2.4 RT‐qPCR

TRIzol™ reagent (#15596026CN, Thermo Fisher) was utilized to extract RNA, and MultiScribe™ reverse transcriptase (#4311235, Thermo Fisher) was utilized to convert total RNA to cDNA. Fast SYBR™ Green Master Mix (#4385610, Thermo Fisher) and the ABI PRISM™ 7900HT Sequence Detection System (ThermoFisher) were used for the qPCR process. Primer sequences were as follows: BICC1 F: 5′‐AAACTGGGCCTGGGCAAATA‐3′, R: 5′‐TTCCAGGAAAGAGGTGCGTG‐3′; NEDD4L F: 5′‐GTGAACAGGGTCCAGAAGCA‐3′, R: 5′‐GAACCACTGAATGACGGGGT‐3′; *β*‐actin F: 5′‐CCCTGGAGAAGAGCTACGAG‐3′, R: 5′‐CGTACAGGTCTTTGCGGATG‐3′. *β*‐actin as internal reference for BICC1 and NEDD4L. Relative expression of genes was calculated using the 2^−∆∆*Ct*^ method.

### 2.5 Western blot (WB)

To extract proteins, cells or tissues were lysed with RIPA lysis buffer (Beyotime, Shanghai, China) enhanced with a protease inhibitor (Roche, Basel, Switzerland) after washed with PBS. The protein concentration was measured using the Beyotime BCA protein kit. Next, SDS PAGE was utilized to separate 30 μg of protein, and the PVDF membrane received the resolved proteins. After the PVDF membrane was closed with 5% BSA for 2 h, the membrane was incubated with primary antibody including BICC1 (sc‐514846, Santa Cruz Biotechnology), E‐cadherin (ab314063, Abcam), Vimentin (ab92547, Abcam), N‐Cadherin (ab76011, Abcam), Snail (MA5‐14801, ThermoFisher), AKT (ab8805, Abcam), p‐AKT (ab38449, Abcam), PI3K (4249, Cell Signaling Technology), p‐PI3K (ab278545, Abcam), NEDD4L (MA5‐32294, ThermoFisher), *β*‐actin (ab8226, Abcam) at 4°C overnight. After a TBST wash the following day, the membrane was incubated for 1 h at room temperature with an HRP‐conjugated secondary antibody (S0001, Affinity, Changzhou, China). Each band was exposed using the Ultra High Sensitivity ECL Kit (#HY‐K1005, Sigma‐Aldrich, St. Louis, Missouri, USA). The intensity of the bands was quantified using Image J analysis software.

### 2.6 Tumor formation in nude mice

Eighteen 6‐week‐old female BALB/c nude mice were housed under specific pathogen‐free (SPF) feeding conditions. The nude mice were randomly divided into three groups (*n* = 6): control, sh‐NC, and sh‐BICC1 groups. Sh‐NC or sh‐BICC1 was transfected into AGS cells. Subsequently, 2 × 10^6^ AGS cells were administered subcutaneously into the mice to induce tumor growth in vivo. Every 5 days following the injection, we measured the tumor volume. The mice were euthanized after 25 days, and the tumors were excised for histological examination and additional research. The study was approved by the Ethics Committee of Hunan University of Medicine.

### 2.7 Immunohistochemistry

Sections of tumor tissue were rehydrated in a graded ethanol solution after being dewaxed. To repair antigens, the dewaxed tissue samples were boiled in 10 mM citrate buffer (pH 6.0) for 20 min. They were then treated with primary antibodies for a night at 4°C after being blocked with goat serum. Subsequently, they were incubated with HRP‐labeled antibodies (1:200, S0001, Affinity) for 2 h. Sections were then re‐stained with hematoxylin after being color‐developed with 3, 3′‐diaminobenzidine (DAB). Primary antibodies used in this part were as follows: anti‐BICC1 (sc‐514846, Santa Cruz Biotechnology), and anti‐E‐Cadherin (ab314063, Abcam).

### 2.8 Clone formation assay

A clone formation assay was performed to measure cell proliferation. Six‐well plates were filled with GC cells at a density of 500 cells per well, and the plates were incubated for 2 weeks at 37°C. After the media was discarded and washed twice with PBS, the cells were then fixed with 4% paraformaldehyde for 15 min. Subsequently, the cells were stained with 0.1% crystal violet. The cloned cells were imaged and quantified using a light microscope (Olympus, Tokyo, Japan).

### 2.9 Wound healing assay

Different groups of GC cells were inoculated into 6‐well plates and when the cells reached approximately 90%, wounds were scratched using a sterile pipette tip. Microscopy was utilized to image the scratch wounds at 0 and 24 h. The cells were grown in a medium devoid of FBS for 24 h and the pace of wound healing or cell migration was calculated as the average distance between the two edges of the cell‐free area.

### 2.10 Transwell invasion assay

The transwell assay was used to assess GC cell invasion capacity. Matrigel polycarbonate membranes were coated in the Transwell chamber. After that, we added medium containing serum to the bottom chamber and 2 × 10^4^ cells in serum‐free medium to the top chamber. The cells were incubated for 24 h at 37°C. Non‐invasive cells at the top of the membrane were removed. 0.1% crystal violet was used to stain the invasive cells at the membrane's bottom after they had been fixed with 4% paraformaldehyde. The number of invasive cells was determined by counting them under a microscope.

### 2.11 Co‐immunoprecipitation (Co‐IP)

The experimental method for Co‐IP was referenced in previous studies.^21^ Cells were lysed using RIPA lysis buffer containing a protease inhibitor and the lysates were sonicated for 30 s before being placed on ice for 1 h. The supernatant was collected and incubated with the anti‐BICC1 (sc‐514846, Santa Cruz Biotechnology) or an IgG negative control (ab172730, Abcam) at 4°C overnight on a rotator. Pretreated Protein A/G magnetic beads (YJ003, Epizyme Biomedical Technology Co., Ltd., Shanghai, China) were added to the residual supernatant and incubated for 3 h at 4°C on a shaker to form antigen–antibody‐magnetic bead complexes. The immunocomplex was washed with washing buffer, and the protein was collected for WB.

### 2.12 Ubiquitination assay

OE‐NC, OE‐NEDD4L, Myc‐NEDD4L, Flag‐BICC1, HA‐K48R, and HA‐K63R were transfected into the GC cells or 293T cells. Then they were incubated with 10 μM MG132 (Sigma‐Aldrich, St. Louis, Missouri, USA) for 6 h. The cells were treated with lysis buffer to produce whole‐cell lysates. For a full night at 4°C, the supernatant was incubated with corresponding amounts of protein A/G beads and anti‐BICC1 (sc‐514846, Santa Cruz Biotechnology). The magnetic beads were washed, and the immunoprecipitation complexes were separated by WB with anti‐HA antibody (#3724, CST), Myc‐Tag (#2272, CST), and Flag‐Tag (#14793, CST).

### 2.13 Protein stability assays

A final concentration of 100 μg/mL cycloheximide (CHX, Sigma‐Aldrich) was added to the culture medium in different groups of cells. Cell lysates were collected at 0, 2, 4, and 6 h after CHX treatment, and BICC1 protein expression was analyzed by WB.

### 2.14 Statistical analysis

The GraphPad Prism software 8.0 (San Diego, CA, USA) was used to analyze the data. Each experiment was performed in triplicate, and the data were presented as the mean ± standard deviation (SD). The significance of comparisons between two groups was evaluated using the Student's *t*‐test, and the presence of statistically significant differences in multiple comparisons was determined using one‐way ANOVA in conjunction with Tukey's post hoc test. Statistical significance was defined as *p* < 0.05.

## 3 RESULTS

### 3.1 Elevated levels of BICC1 were found in GC tissue samples and cell lines

**FIGURE 1:**
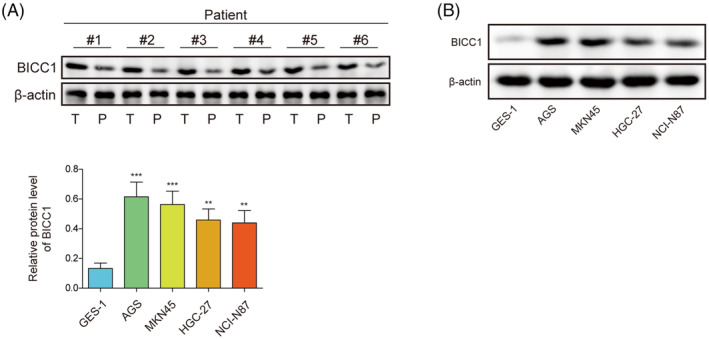
Elevated levels of BICC1 were found in GC tissue samples and cell lines. (A) WB was used to determine BICC1 protein expression in patients with GC tumors (T: tumor tissue, P: para‐carcinoma tissue). (B) WB was used to evaluate the protein expression of BICC1 in GES‐1 and GC cell lines. *p < 0.05, **p < 0.01, ***p < 0.001.

There were 30 pairs of GC tissues and corresponding para‐cancerous normal tissues were collected for the study to investigate the expression of BICC1. The results indicated that the protein levels of BICC1 were expressed higher in GC tissues compared to para‐cancerous normal tissues (Figure 1A). In addition, the expression of BICC1 in GC cell lines was examined. Compared to human gastric mucosa cells (GES‐1), we found that GC cell lines (AGS, MKN45, HGC‐27, NCI‐N87) exhibited elevated levels of BICC1 expression (Figure 1B). Additionally, we also analyzed the ubiquitination level of BICC1 in GES‐1 and GC cell lines. The results showed that the ubiquitination level of BICC1 was higher in GES‐1 than in GC cell lines (Figure S1A). In summary, the results presented above showed that GC tissues and GC cell lines both have considerable expression of BICC1, indicating a potential role for BICC1 in the control of GC processes.

### 3.2 Knockdown of BICC1 inhibited GC tumor growth and PI3K/AKT‐mediated EMT

**FIGURE 2:**
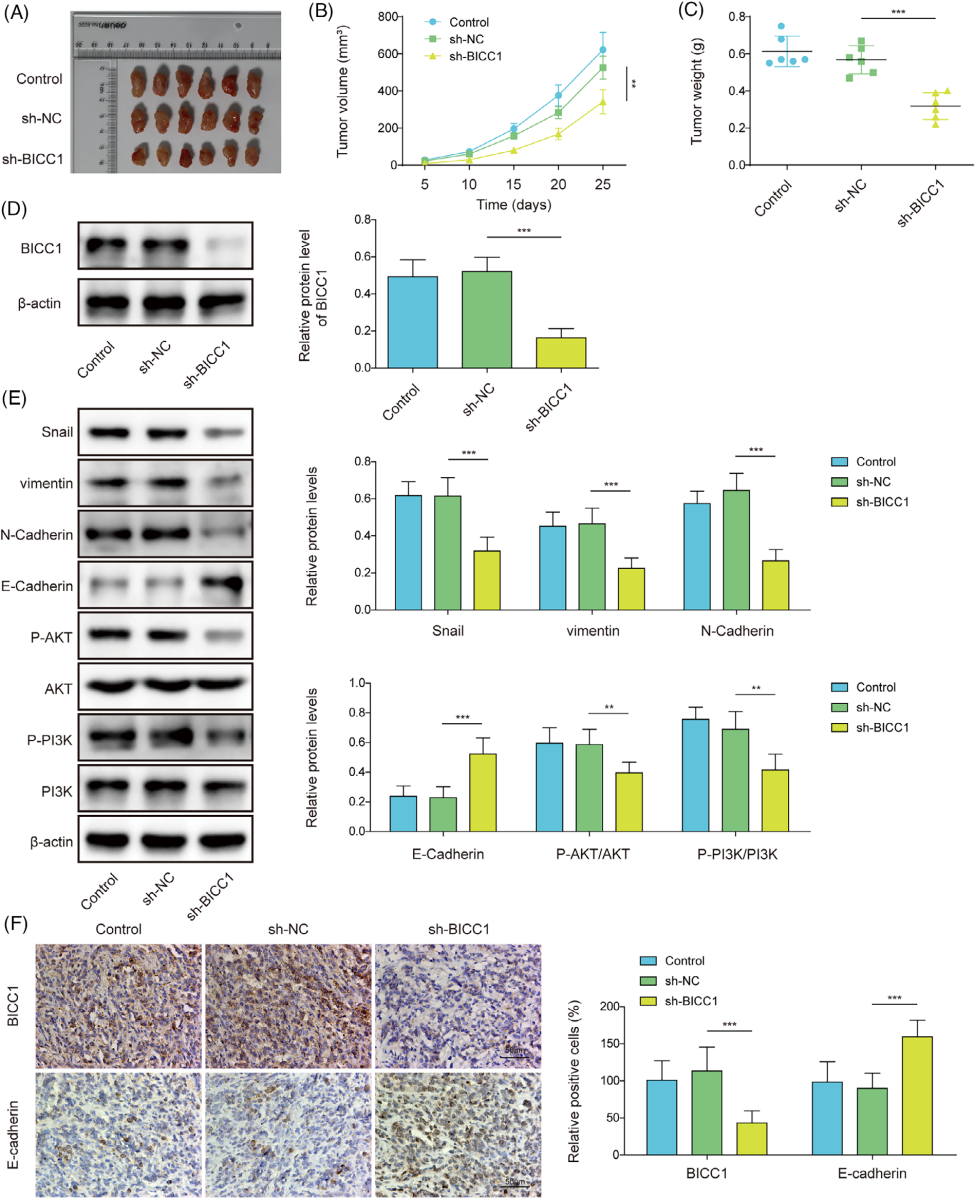
Knockdown of BICC1 inhibited GC tumor growth and PI3K/AKT‐mediated EMT. BICC1 knockdown AGS cells or control cells were injected into the flank region of nude mice to construct a xenograft mode. Nude mice were divided into three groups based on the cells injected into the mice: Control, sh‐NC, and sh‐BICC1 (*n* = 6). (A) Representative images of tumors isolated from xenograft tumor models. (B) Comparison of tumor volume in different groups of nude mice at each time point. (C) Comparing the tumor weights of several mouse groups. (D) WB was used to evaluate the protein expression level of BICC1 in tumor tissues of different mice groups. (E) WB revealed the protein expression of Snail, vimentin, N‐Cadherin, E‐Cadherin, p‐AKT, AKT, PI3K, and p‐PI3K in the tumor tissues of several mouse groups. (F) IHC was used to identify the expression of BICC1 and E‐Cadherin in the tumor tissues of various mouse groups. *p < 0.05, **p < 0.01, ***p < 0.001.

We created a xenograft model by subcutaneously injecting sh‐BICC1 AGS cells or control cells into nude mice to investigate the role of BICC1 in vivo. Tumor size and volume were significantly reduced in the sh‐BICC1 group compared with the sh‐NC group (Figure 2A, B). Furthermore, the downregulation of BICC1 resulted in a decrease in the tumor weight (Figure 2C). WB was performed to examine BICC1 expression in tumor tissues, revealing that the sh‐BICC1 group's tumor tissues exhibited lower BICC1 levels (Figure 2D). We also assessed EMT indicators and found that sh‐BICC1 down‐regulated the levels of vimentin, snail, and N‐Cadherin. Conversely, E‐Cadherin expression was significantly higher in the sh‐BICC1 group when compared to the sh‐NC groups (Figure 2E). We investigated whether BICC1 influenced the PI3K/AKT pathway, which is known to be involved in the EMT process in malignancies. Our results discovered that p‐PI3K and p‐AKT levels were suppressed following BICC1 knockdown (Figure 2E), indicating that the PI3K/AKT was less active in the absence of BICC1. Finally, IHC was employed to validate the expression levels of BICC1 and E‐Cadherin on tumor tissues. As expected, the findings demonstrated that BICC1 suppression enhanced E‐Cadherin expression. (Figure 2F). In summary, the results imply that BICC1 deletion impedes GC growth and EMT in vivo through the PI3K/AKT signaling pathway.

### 3.3 Knockdown of BICC1 negatively regulated proliferation, migration, invasion, and PI3K/AKT signaling pathway in GC cells

**FIGURE 3:**
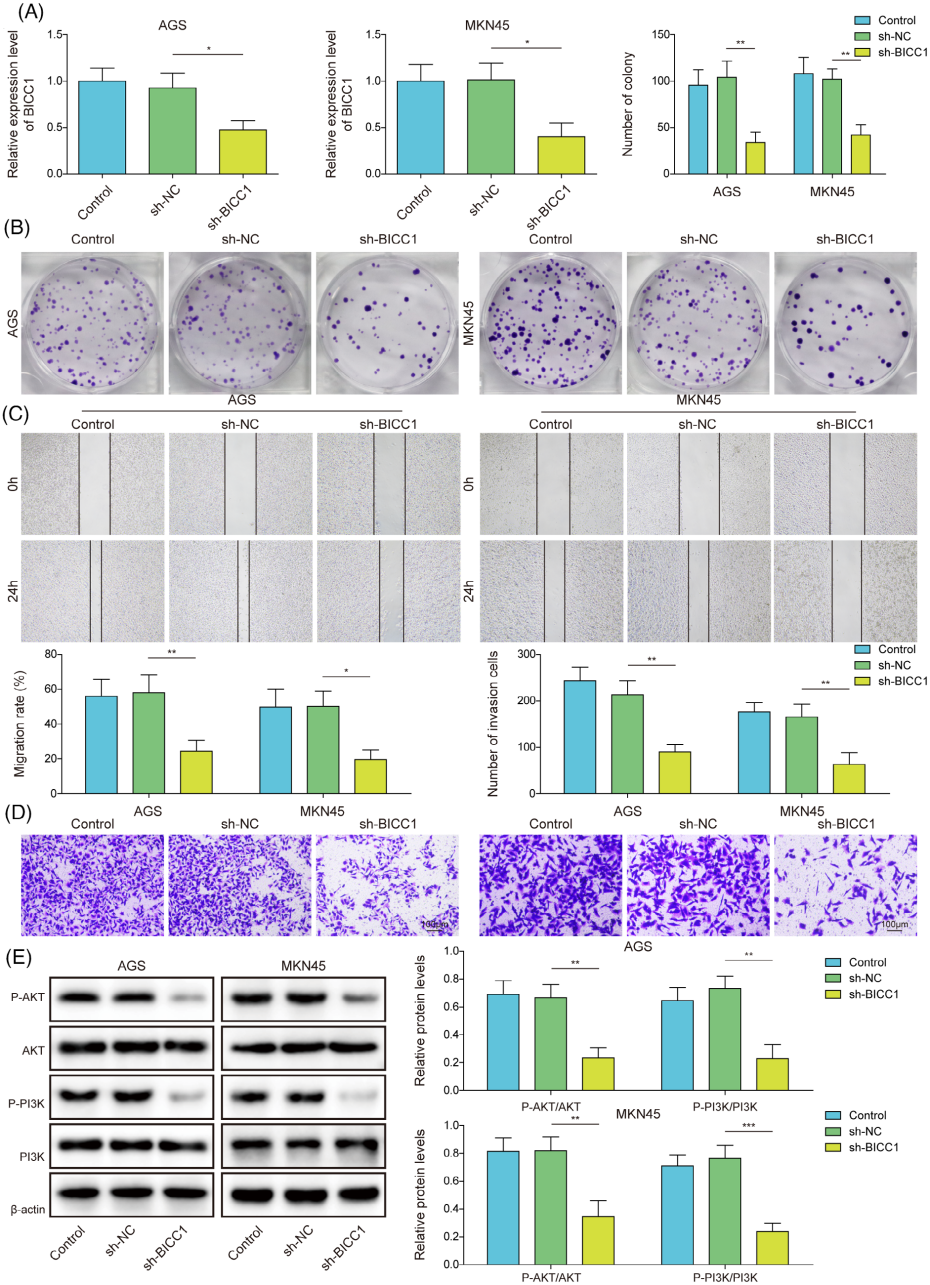
Knockdown of BICC1 negatively regulated proliferation, migration, invasion, and PI3K/AKT signaling pathway in GC cells. (A) RT‐qPCR was utilized to measure the BICC1 mRNA expression in GC cells that transfected with either sh‐BICC1 or sh‐NC. (B) The clone formation assay was performed to evaluate the proliferation of GC cells transfected with sh‐BICC1 or sh‐NC. (C) GC cells transfected with sh‐BICC1 or sh‐NC were used the wound healing test to measure migration ability. (D) By using the transwell test, the invasion potential of GC cells transfected with sh‐BICC1 or sh‐NC was identified. (E) WB identified PI3K, p‐PI3K, AKT, and p‐AKT protein expression in GC cells transfected with sh‐BICC1 or sh‐NC. *p < 0.05, **p < 0.01, ***p < 0.001.

Additionally, we confirmed that BICC1 is active in MKN45 and AGS cells. RT‐qPCR was initially employed to assess the efficiency of BICC1 knockdown, revealing that GC cells transfected with sh‐BICC1 significantly down‐regulated the mRNA expression of BICC1 (Figure 3A). However, there was no discernible difference in BICC1 expression between the transfected sh‐NC group and the control group. This finding suggests that BICC1 has been effectively knocked down in GC cells. Subsequently, we discovered that the ablation of BICC1 reduced the clonal number of GC cells (Figure 3B). Furthermore, when compared to control and sh‐NC groups, BICC1 knockdown diminished the cells' capacity for migration and invasion (Figure 3C, D). BICC1‐silenced cells exhibited decreased phosphorylation of both AKT and PI3K, which hindered the PI3K/AKT pathway (Figure 3E). These findings indicate that the knockdown of BICC1 possesses anticancer activity. BICC1 silencing may prevent GC cells from proliferating, migrating, invading, and PI3K/AKT activation.

### 3.4 Knockdown of BICC1 inhibited GC cell migration and invasion by suppressing PI3K/AKT‐mediated EMT

**FIGURE 4:**
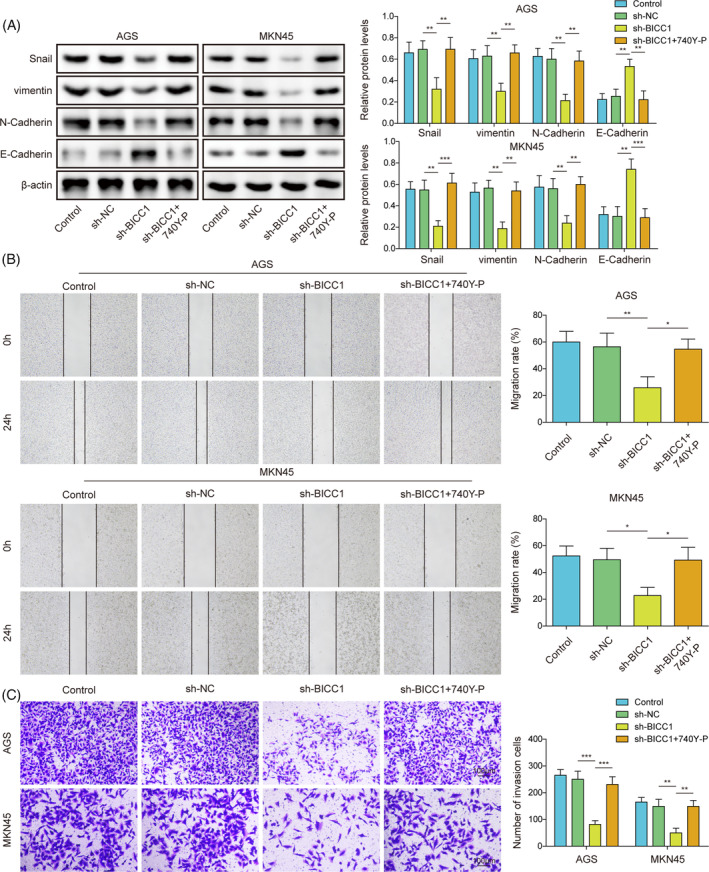
Knockdown of BICC1 inhibited GC cell migration and invasion by suppressing PI3K/AKT‐mediated EMT. Sh‐BICC1 or sh‐NC was transfected into AGS and MKN45 to silence the BICC1 gene. For the agonist‐treated group, AGS and MKN45 were pretreated with the 20 μM PI3K/Akt agonist 740Y‐P for 24 h before transfection of shRNA. (A) The protein expression of E‐Cadherin, N‐Cadherin, vimentin, and Snail in GC cells was detected by WB. (B) Wound healing assay was used to detected the migration ability of GC cells. (C) Transwell assay was used to detected invasion ability of GC cells. *p < 0.05, **p < 0.01, ***p < 0.001.

To examine the mechanism by which BICC1 contributes to GC through the regulation of the PI3K/AKT pathway, we conducted rescue tests by administering the PI3K/AKT agonist 740Y‐P. Compared to the sh‐NC groups, BICC1 knockdown increased the expression of E‐Cadherin while decreasing the expression of Snail, N‐Cadherin, and vimentin. Notably, 740Y‐P counteracted the inhibitory effect of BICC1 knockdown on EMT (Figure 4A). Additionally, GC cells' migration and invasion features were reduced by sh‐BICC1. However, treatment with 740Y‐P reversed the downregulation of migration and invasion abilities induced by sh‐BICC in GC cells (Figure 4B, C). In summary, our findings demonstrate that the ablation of BICC1 suppresses GC cell migration and invasion by inhibiting PI3K/AKT‐mediated EMT.

### 3.5 NEDD4L expression was reduced in GC, and NEDD4L mediated ubiquitination of BICC1

**FIGURE 5:**
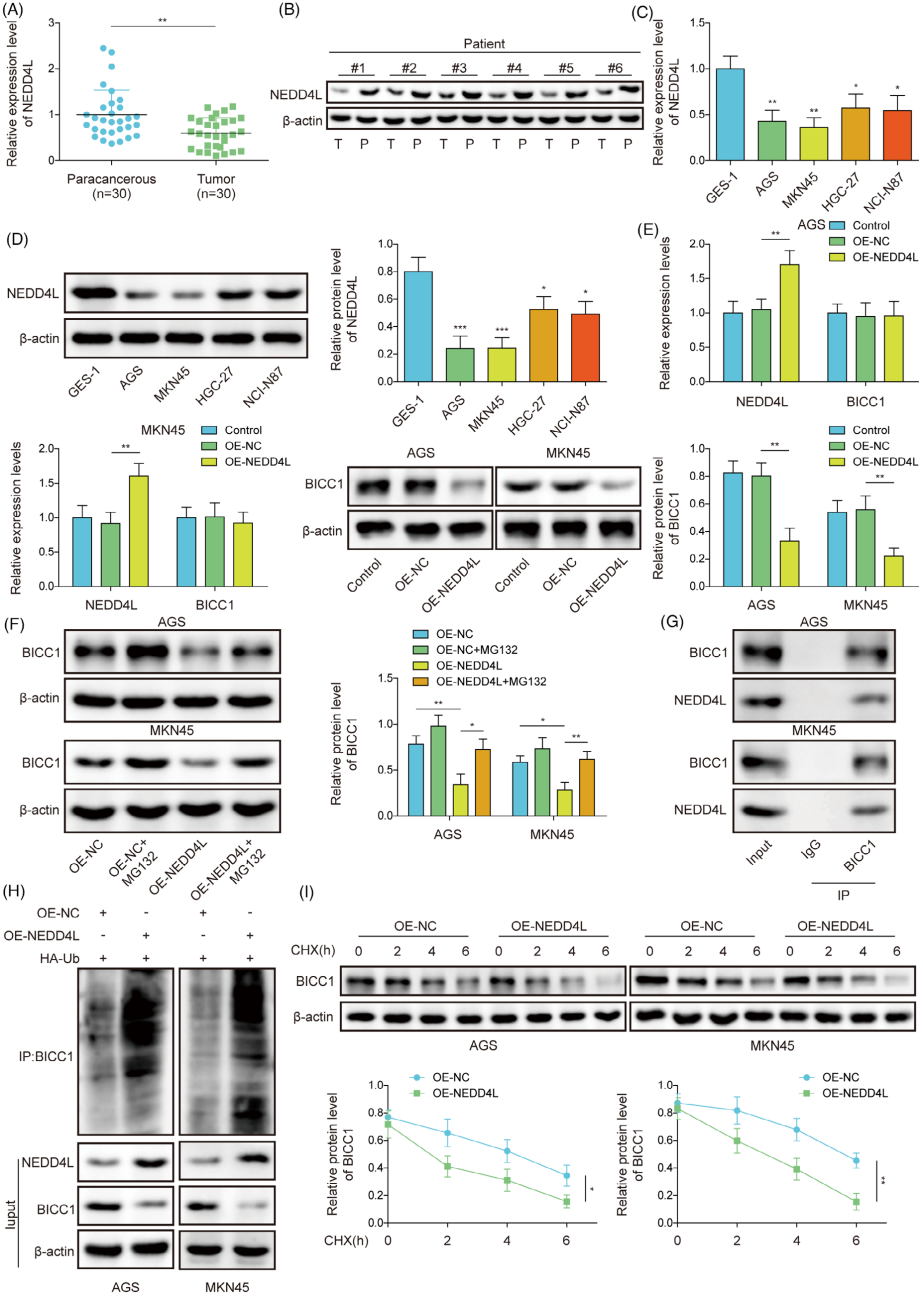
NEDD4L expression was reduced in GC, and NEDD4L mediated ubiquitination of BICC1. (A) RT‐qPCR was used to determine the mRNA expression level of NEDD4L in patients with GC. (B) WB determined the NEDD4L protein expression level in patients with GC tumors. (C) RT‐qPCR was used to determine the BICC1 mRNA expression level in GES‐1 and GC cell lines. (D) WB was used to assess BICC1 protein expression in GES‐1 and GC cell lines. (E) WB and RT‐qPCR were used to identify NEDD4L and BICC1 expression in GC cells transfected with OE‐NEDD4L. (F) WB determined the BICC1 protein expression in GC cells with OE‐NEDD4L, OE‐NC, or MG132. (G) Co‐immunoprecipitation (Co‐IP) assay was used to evaluate the interaction between NEDD4L and BICC1. (H) OE‐NEDD4L or OE‐NC was transfected into AGS and MKN45 cells and co‐transfected with HA‐Ub. Co‐IP binding WB was used to extract BICC1 and ubiquitin antibody labels to detect ubiquitination. (I) Using the cycloheximide (CHX) chase assay, the impact of NEDD4L on BICC1 stability was assessed. *p < 0.05, **p < 0.01, ***p < 0.001.

We investigated the upstream regulatory pathways of BICC1 to elucidate underlying mechanism of its regulation. It was found that the tumor tissues from GC patients had significantly reduced levels of NEDD4L mRNA and protein (Figure 5A, B). Additionally, we observed that NEDD4L expression was downregulated in GC cell lines, exhibiting similar outcomes (Figure 5C, D). Importantly, it was found that the protein expression of NEDD4L and BICC1 was negatively correlated in GC (Figure S1B). NEDD4L was predicted to ubiquitinate modification of BICC1, and thus the molecular mechanism of NEDD4L on BICC1 was also explored. By transfecting the OE‐NEDD4L vector into GC cells, an overexpression model was established. The results indicated that transfection of the vector significantly enhanced the expression of NEDD4L (Figure 5E). Interestingly, while NEDD4L overexpression suppressed BICC1 protein expression, it did not affect the RNA level (Figure 5E). Therefore, we speculate that NEDD4L mediates the degradation of BICC1 rather than altering its transcription.

To verify whether NEDD4L degraded BICC1 through the protease system, the proteasome inhibitor MG132 was used to treat GC cells. MG132 overrode NEDD4L overexpression's inhibitory impact on BICC1 expression (Figure 5F), further confirming that the degradation of BICC1 was mediated by NEDD4L via the protease system. Moreover, Co‐IP experiments showed that the NEDD4L protein interacted with the BICC1 protein (Figure 5G). OE‐NEDD4L raised BICC1's ubiquitination level compared to the OE‐NC group (Figure 5H). In addition, we administered CHX, a protein synthesis inhibitor, to both OE‐NC and overexpressing NEDD4L cells and we discovered that overexpressing NEDD4L cells had a considerably shorter BICC1 protein half‐life than OE‐NC cells (Figure 5I). These results demonstrate that NEDD4L accelerates the degradation of BICC1. Furthermore, we also explored the effect of NEDD4L on the protein ubiquitination pattern of BICC1. The results indicated that overexpression of NEDD4L increased the levels of K48‐linked ubiquitination of BICC1, while there was no significant change in the levels of K63‐linked ubiquitination (Figure S1C). Subsequently, using HA‐tagged ubiquitin mutants (K48R and K63R), we found that the level of BICC1 ubiquitination was significantly reduced after the K48 mutation, confirming that NEDD4L mediates K48‐linked ubiquitination of BICC1 (Figure S1D). In summary, our findings demonstrate that NEDD4L facilitates BICC1 protein breakdown through K48‐linked ubiquitination modification of BICC1.

### 3.6 Through the suppression of BICC1 expression, NEDD4L overexpression prevented GC cell proliferation, migration, invasion, and EMT

**FIGURE 6:**
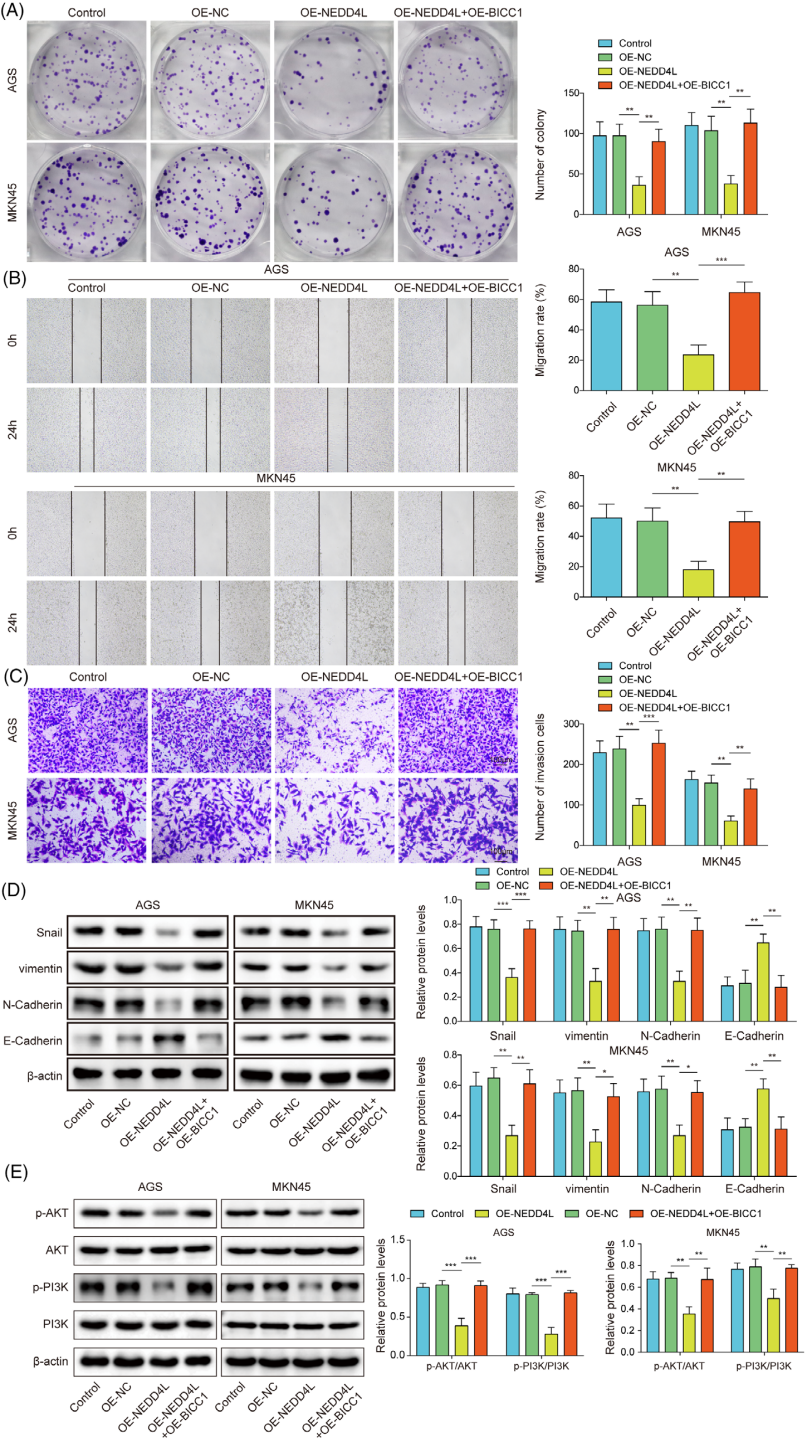
Through the suppression of BICC1 expression, NEDD4L overexpression prevented GC cell proliferation, migration, invasion, and EMT. AGS and MKN45 cells were transfected with OE‐NC or OE‐NEDD4L or co‐transfected with OE‐NEDD4L and OE‐BICC1 for rescue experiments. (A) The proliferation of GC cells was measured using a clone formation assay. (B) The wound healing test was used to measure migration ability. (C) The invasion potential of GC cells was evaluated using a Transwell assay. (D) WB was performed to determine the protein expression of N‐Cadherin, Snail, vimentin, E‐Cadherin. (E) WB was used to determine the protein expression of PI3K, p‐PI3K, AKT, and p‐AKT in GC cells. *p < 0.05, **p < 0.01, ***p < 0.001.

To provide additional insights into the function of the NEDD4L/BICC1 axis in GC, we transfected GC cells with either OE‐NEDD4L or OE‐BICC1. First, we examined the effect on BICC1 ubiquitination levels after the simultaneous overexpression of NEDD4L and BICC1. Result demonstrated that there was no significant effect on the ubiquitination level of BICC1 after this simultaneous overexpression; however, the protein level of BICC1 was significantly increased (Figure S1E). This elevated BICC1 protein level was primarily attributed to the transfection of OE‐BICC1. The test for clone formation revealed that elevating NEDD4L impeded the proliferation of cells. While simultaneously increasing BICC1 expression reversed the proliferation inhibition induced by overexpression of NEDD4L (Figure 6A). In addition, the invasion and migration capacities of GC cells was suppressed by overexpression of NEDD4L. However, the inhibitory effect of overexpressed NEDD4L on the invasion and migration capacities of GC cells was reversed by upregulating BICC1 expression (Figure 6B, C). The EMT markers were also evaluated, and we discovered that upregulating NEDD4L resulted in a rise in E‐Cadherin protein expression and a fall in N‐Cadherin, vimentin, and snail protein expression. Increasing BICC1 expression reversed this phenomenon (Figure 6D). To further explore the involvement of PI3K/AKT in BICC1‐mediated EMT, we examined the expression of this pathway. It was found that the activation of the PI3K‐AKT pathway was inhibited following the overexpression of NEDD4L alone. In contrast, the simultaneous overexpression of NEDD4L and BICC1 activated the PI3K‐AKT pathway, which mitigated the effects observed with the overexpression of NEDD4L alone (Figure 6E). These results emphasize that the anticancer potential exerted by NEDD4L in GC is achieved by negatively regulating the expression of BICC1.

## 4 DISCUSSION

GC is one of the most prevalent and lethal cancers of the digestive system.^22^, ^23^ Despite the availability of more diagnostic and treatment options for GC than ever before, the disease's tendency to metastasize leads to GC recurrence and a poor prognosis. Recent research indicates that the fundamental processes governing the metastatic spread of GC remain poorly understood. Therefore, investigating the pathophysiology of GC and exploring potential intervention options could significantly improve the prognosis of the disease. This investigation primarily focused on the roles that potential therapeutic targets in GC.

In this study, BICC1 expression was elevated in GC cell lines and patients. The findings of this study is consistent with earlier research that discovered that GC exhibited high levels of BICC1.^11^ These findings showed a strong relationship between BICC1 and the growth or metastasis in GC. Subsequently, we also verified the specific function of BICC1 in GC and discovered that GC migration and proliferation were inhibited both in vitro and in vivo by BICC1 deletion. Numerous studies have consistently found that BICC1 could promote proliferation and migration in solid tumors. In pancreatic cancer, BICC1 promoted resistance of pancreatic cancer to chemotherapeutic agents and increased the stemness character of the tumor.^24^ Proliferation and metastatic progression of ovarian cancer could be promoted by recruiting BICC1.^25^ The results of our study agree with this research, demonstrating that BICC1 has pro‐carcinogenic potential. Additionally, ubiquitination level of BICC1 was higher in GES‐1 than in GC cell lines, whereas there was no statistically significant difference in the ubiquitination level in GC cell lines. This suggests that the differential expression of BICC1 in GC may be influenced by ubiquitination modifications. Additionally, several factors contribute to the differential expression of BICC1 in gastric cancer, including other post‐translational modifications such as acetylation and transcriptional regulation. For instance, miR‐101‐3p and miR‐199b‐5p promote apoptosis in oral cancer cells by targeting and inhibiting BICC1 expression.^9^ Cis‐regulatory elements within intron 3 of the BICC1 gene regulate the activity of the BICC1 promoter region, thereby inducing BICC1 transcription.^26^ The reasons for the differences in ubiquitination levels of BICC1 in different cell lines require further investigation.

The complex process of tumor metastasis involves the interplay of various proteins and signaling pathways. Recent research has demonstrated that EMT is a critical stage in metastasis and is associated with a poor prognosis for individuals with GC.^27^, ^28^ EMT is responsible for the majority of human malignancies, as it enables cancer cells to acquire the ability to migrate and invade while losing their polarity and intercellular adhesion.^29^, ^30^ In this study, we found that BICC1 silencing blocked the EMT process in GC. Although previous studies have suggested a relationship between BICC1 and EMT, our findings provide experimental support for this connection. Through bioinformatics analysis, Meng and colleagues found that BICC1 was predominantly enriched in the EMT pathway and speculated that it plays a role in the lymphatic metastasis of pancreatic cancer.^12^ Our current work corroborates Meng's hypothesis, demonstrating a strong association between BICC1 and the EMT process.^12^ Furthermore, the PI3K/AKT signaling pathway is a traditional pro‐oncogenic mechanism, which is closely related to the EMT process.^31^ Previous research shown that the PI3K/AKT may cause epithelial indicators to be downregulated, whereas mesenchymal markers and transcription factors unique to EMT were upregulated.^32^, ^33^ Our study found that PI3K/AKT signaling pathway inactivation was brought about by BICC1 knockdown. Furthermore, we showed that BICC1 induced the EMT process by activating the PI3K/AKT. According to earlier studies, the PI3K/AKT pathway is essential to EMT.

To deeply investigate the molecular mechanism of BICC1, the upstream mechanisms regulating BICC1 expression were also explored in this study. In GC tissues and cells, NEDD4L was shown to be lower expressed. Proliferation, migration, and EMT of GC cells were reduced by increased NEDD4L. Several researchers also suggested that NEDD4L downregulation was strongly correlated with cancer development in various types of solid tumors. For example, NEDD4L could inhibit pancreatic cancer proliferation and metastasis through suppressing the protein ANXA2.^34^ In renal clear cell carcinoma, NEDD4L overexpression significantly reduced malignant manifestations such as cell proliferation and migration.^35^ The present study's findings consistent with the NEDD4L findings reported in the literature, indicating that NEDD4L functions as a tumor suppressor gene. Through the ubiquitin‐proteasome pathway, NEDD4L, an E3 ubiquitin ligase, promotes the degradation of many proteins.^36^, ^37^ Researchers discovered that in lung cancer, NEDD4L increased Notch2 ubiquitination and degradation.^38^ NEDD4L also induced MEKK2 ubiquitination to inhibit inflammation.^39^ Consistent with these studies, the current study also found that NEDD4L could inhibit tumor progression in GC by inducing degradation of BICC1 protein through the ubiquitin‐proteasome pathway. Furthermore, previous studies have also found that NEDD4L increases the K48‐linked ubiquitination of the target protein TRAF3, leading to a decrease in the protein level of TRAF3.^40^ Consistent with previous studies, in this study, we have alse demonstrated that NEDD4L reduced BICC1 protein expression through a K48‐linked ubiquitination pattern.

Although the present study proposed a new mechanism to regulate the EMT process of GC, it still had a slight drawback. On the one hand, confirmation that BICC1 and NEDD4L expression is connected to cancer metastasis requires a substantial number of clinical samples. On the other hand, the specific site where NEDD4L regulates the ubiquitination of BICC1 protein also needs to be identified in future studies.

In summary, this study demonstrated that NEDD4L inhibits GC proliferation, migration, and PI3K/AKT‐mediated EMT through the ubiquitination of BICC1. Therefore, our analysis suggests potential targets for GC therapy and provides a theoretical foundation for the roles of BICC1 and NEDD4L in GC.

## CONFLICT OF INTEREST STATEMENT

The authors declare that they have no conflict of interest.

## ETHICS STATEMENT

Written informed consent was provided by every patient. And the study was approved by the Ethics Committee of Hunan University of Medicine. The Hunan University of Medicine Ethics Committee gave its approval for this study.

## CONSENT FOR PUBLICATION

The informed consent obtained from study participants.

## Supporting information


**FIGURE S1.** Investigation of the mechanism of BICC1 ubiquitination. (A) Co‐IP binding WB was used to detect ubiquitin of BICC1 in GES‐1 and gastric cancer cell lines (AGS, MKN45, HGC‐27, NCI‐N87). (B) Protein correlation analysis between NEDD4L and BICC1 at GC. (C) Co‐IP binding WB was used to detect K48/K63 linked ubiquitin of BICC1 in AGS and MKN45 cells. (D) Co‐IP binding WB was used to detect ubiquitin of BICC1 in GC cells co‐transfected with Myc‐NEDD4L, Flag‐BICC1, HA‐K48R and HA‐K63R. (E) Co‐IP binding WB was used to detect ubiquitin of BICC1 in GC cells e transfected with OE‐BICC1 or OE‐NEDD4L.

## Data Availability

Data sharing not applicable to this article as no datasets were generated or analysed during the current study.
