# Supplementary figures and images for: NEDD4L inhibits epithelial‐mesenchymal transition in gastric cancer by mediating BICC1 ubiquitination
Source: Kaohsiung J Med Sci. 2024 Dec 24;41(2):e12924. doi: 10.1002/kjm2.12924 (PMC11827545; doi:10.1002/kjm2.12924)

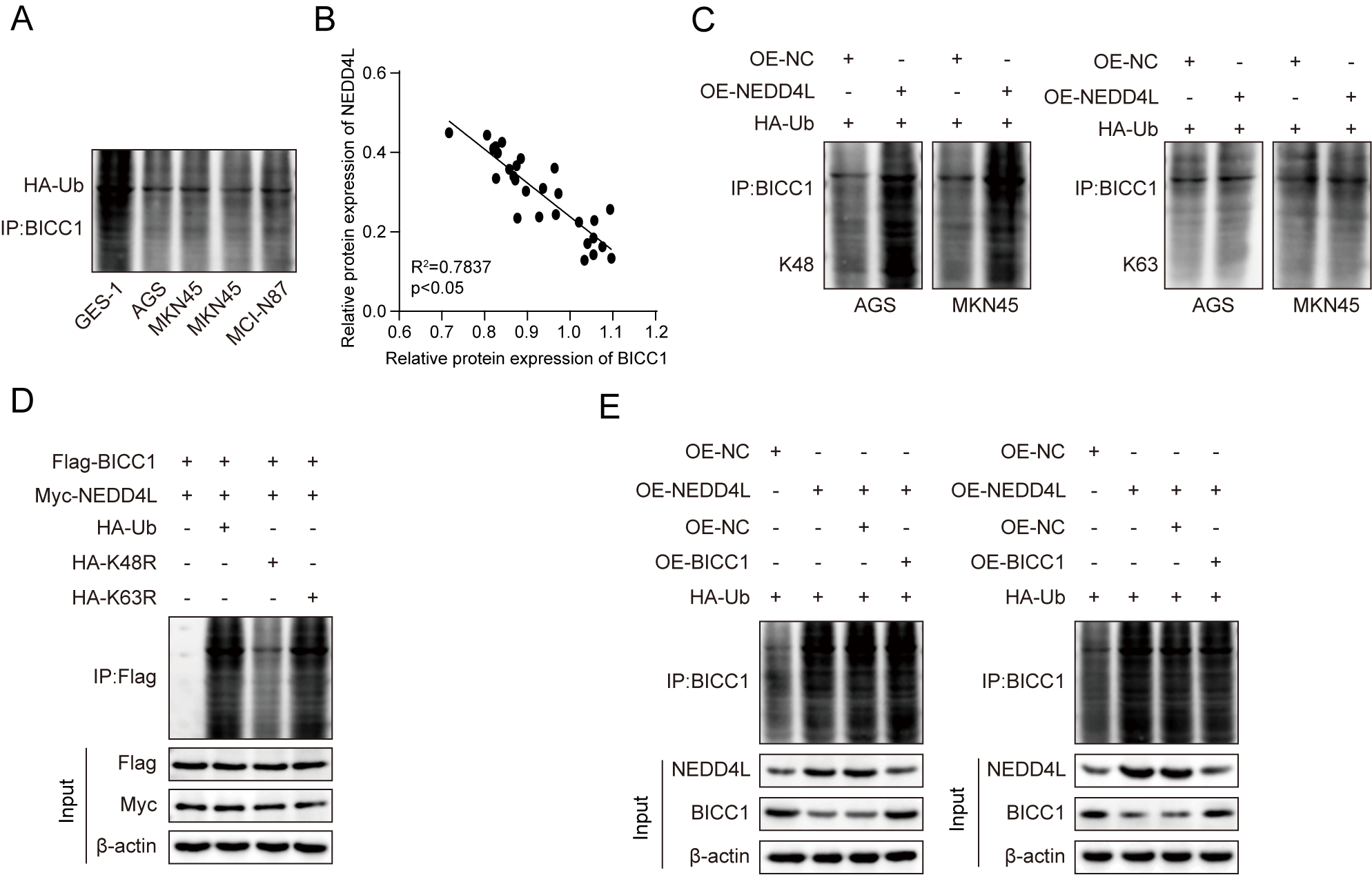

Supplement: Supplementary file 1 — FIGURE S1. Investigation of the mechanism of BICC1 ubiquitination. (A) Co‐IP binding WB was used to detect ubiquitin of BICC1 in GES‐1 and gastric cancer cell lines (AGS, MKN45, HGC‐27, NCI‐N87). (B) Protein correlation analysis between NEDD4L and BICC1 at GC. (C) Co‐IP binding WB was used to detect K48/K63 linked ubiquitin of BICC1 in AGS and MKN45 cells. (D) Co‐IP binding WB was used to detect ubiquitin of BICC1 in GC cells co‐transfected with Myc‐NEDD4L, Flag‐BICC1, HA‐K48R and HA‐K63R. (E) Co‐IP binding WB was used to detect ubiquitin of BICC1 in GC cells e transfected with OE‐BICC1 or OE‐NEDD4L. [file KJM2-41-e12924-s001.tif]
